# High pressure excursion in a radial design oxygenator

**DOI:** 10.1051/ject/2024019

**Published:** 2024-12-20

**Authors:** Ashley Svec, Tyler Eadie, Brandon D’Aloiso, Peter Arlia

**Affiliations:** 1 UPMC Presbyterian Perfusion 200 Lothrop Street Pittsburgh PA 15213 USA

**Keywords:** Cardiopulmonary bypass, High pressure excursion, Case report

## Abstract

Oxygenator high-pressure (HPE) is a phenomenon that can occur during cardiopulmonary bypass (CPB) in which the oxygenator inlet pressure increases rapidly, thereby limiting flow. Most perfusionists globally do not use inlet oxygenator pressure monitoring and therefore HPE is not often recognized. HPE may occur for various reasons, and it is not fully understood. Patient factors that put a patient at a higher risk of HPE are increased body surface area, blood type, and hematocrit count. Patient size, blood flow, and pressure drops of the oxygenator incorporated into the circuit can also increase the probability of an HPE occurring. This case study overviews our experience when dealing with an interesting case of HPE and the most up-to-date knowledge on appropriate steps to mitigate the effects on the patient.

## Overview

Oxygenator high-pressure excursion (HPE) is a phenomenon that can occur during cardiopulmonary bypass (CPB) in which the oxygenator inlet pressure increases rapidly, thereby limiting flow. The mechanism of HPE is not fully understood, but the literature describes aggregation of platelets and swelling of oxygenator fibers to be possible causes [1]. HPE can lead to the necessity of an oxygenator changeout while on CPB due to this limited flow. This limited flow may go unnoticed if there is no oxygenator inlet pressure monitoring; however, less than 10% of perfusionists have inlet pressure monitoring incorporated into their circuit [2]. Patients with a large body surface area (BSA), high hematocrit, acute or emergent surgery, and a history of stroke are known to be at a higher risk for HPE during CPB [3]. Blood types have also been seen to affect the possibility of an HPE. A study conducted by Myers et al. [4], tested the blood of 31 patients who had an HPE occurring on CPB; it was concluded that 51.6% of patients with O-positive blood had experienced HPE. In cases where HPE occurs, many temporizing measures can be taken such as additional anticoagulation, hemodilution, Epoprostenol administration and, in unresolving cases, oxygenator changeout [3]. The Medtronic Affinity Fusion Oxygenator used features a Balance^TM^* Biosurface coating that is a hydrophilic polymer coating without heparin for cardiopulmonary bypass circuit devices that reduces platelet adhesion and activation and preserves platelet function. The Terumo FX25 features the Terumo X-Coating which is an albumin-based coating. Currently, no albumin is added to the prime at our center. Much of the literature surrounding HPE discusses cases in which roller pumps were used and therefore describe pressure measurements 500 mmHg. Contemporary centrifugal-based CPB systems do not generate pressures this high during normal operating conditions and therefore have a different set of considerations when diagnosing and treating HPE but lessons from roller pumps are nonetheless important to understanding this phenomenon. In addition, there is literature surrounding HPE with trans-oxygenator pressures such as the study conducted by Fisher et al. [5]. The HPE in this incidence is unique due to the use of the centrifugal CPB system and radial-designed oxygenator.

## Description

A 46-year-old male presented to the operating room at UPMC Presbyterian on October 30, 2023, with an ascending aortic aneurysm. The patient was found to have a calcified bicuspid aortic valve, aortic regurgitation, and hypertension. An aortic valve replacement, hemiarch replacement, and aortic root replacement with deep hypothermic cardiac arrest (DHCA) and retrograde cerebral perfusion were planned. The patient’s blood type was O-positive. Pre-operative laboratory tests were within normal limits: hematocrit 41.5%, prothrombin time (PT) 14 s, APTT 32 s, platelets 246,000/ mL, BUN 17/dL, and creatinine 0.8/dL. The patient’s body surface area was 3.1 m^2^, and the calculated minimal flow to provide adequate perfusion was 6.2 L/min at a cardiac index of 2.0 L/min/m^2^. The patient’s home medications were metoprolol, empagliflozin, and spironolactone. The patient was allergic to Entresto and bee stings. Due to our patient’s larger size, we investigated the patient’s lean body mass to verify our oxygenator would support the patient’s needs. The calculated lean body mass for the patient was 105 kg. Using this to calculate our BSA we find a BSA of 2.3 m^2^ and our calculated indexes to **2.0**: 4.6 L/min, **2.5**: 5.8 L/min, **3.0:** 7.0 L/min verifying that our single oxygenator setup should be able to support the patient.

Before patient induction, the baseline blood gas was pH 7.37, PCO2 44.5 mmHg, PO2 383 mmHg, bicarbonate 25.5 mmol/L, base −0.1, oxygen saturation 100%, hemoglobin 13.9 g/dL, hematocrit 41.5%, sodium 141 mMol/L, potassium 4.2 mMol/L, calcium 1.13 mMol/L, glucose 97 mg/dL, and lactate 0.7 mg/dL. Baseline ACT 125 at 0755. The anesthesia team ran a thromboelastogram (TEG) at 0755 and the platelet count was 214,000/ mL. The baseline TEG had a max amplitude of 75 mm indicating a borderline hypercoagulable state. A Medtronic Affinity Fusion Oxygenation System was used for perfusion in this case (Medtronic Inc., Minneapolis MN). Our CPB circuit consisted of LivaNova S5 HLM, Quest MPS3ND, Medtronic Affinity Fusion hardshell Venous Reservoir, Medtronic Affinity Fusion Adult Oxygenator, a Medtronic custom tubing pack comprises the remainder of the circuit that is coated with a Balance Biosurface coating. The pump was primed with 1400 mL of Plasmalyte-A, before initiating bypass a retrograde autologous prime was performed leaving only about 900 mL of Plasmalyte-A still in the circuit. To that final circulating volume, 5000 units of heparin and 25 g of 20% mannitol are added. The Anesthesia team gave 4000 units of heparin. The post-heparin ACT 444 at 0825. Cardiopulmonary bypass was initiated at 0842 and the patient was cooled for 30 min. The cooling gradient was maintained in the safe range always less than 10 °C as is standard practice at our center. The patient’s starting bladder temp was 36.2 °C and it was cooled to 29.5 °C at the time of circ arrest. The revolutions per minute of the pump (RPM) were maximized at 3300, with a measured flow of 6.1 L/min and an arterial resistance of 248 mmHg. The perfusion team was mindful to keep the FIO_2_ set to 100% due to the patient’s increased oxygenation requirements. After eight minutes on bypass, a blood gas was drawn to determine adequate oxygenation and lab values; pH 7.30, PCO2 50.7 mmHg, PO2 394 mmHg, bicarbonate 24.1 mmol/L, base −1.4, oxygen saturation 100%, hemoglobin 11.6 g/dL, hematocrit 35.5%, sodium 136 mMol/L, potassium 7.5 mMol/L, calcium 1.00 mMol/L, glucose 146 mg/dL, and lactate 1.1 mg/dL. The hyperkalemia was due to running the arterial blood gas immediately after the initial dose of cardioplegia. Cardioplegia was given according to our standard microplegia protocol. Our protocol calls for a 1200 mL blood-based cardioplegia initial dose. Added to the blood by way of a Quest MPS3 was 40 mEq/L of Potassium Chloride and 4 mEq/L of Magnesium Sulfate. At this time, the venous blood return temperature was 31.4 °C and the radial arterial blood pressure was measured at 65 mmHg. With the RPMs stable at 3500, the flow had decreased to 3.9 L/min with a post-oxygenator arterial resistance of 153 mmHg, and it was observed that flow to the patient was below the calculated minimal flow. Because RPMs were set to the maximum value, flow could not be increased, and the perfusion team began troubleshooting with the assistance of the surgeon and anesthesiologist. Troubleshooting began with ensuring the centrifugal head was seated properly. To verify proper placement, the position was verified at a point during the aortic repair when the surgeon instructed to decrease flow. The perfusion team decreased the RPMs to 0 RPMs to disengage and reengage the pump head to confirm proper placement. Our circuit features a Medtronic Affinity CP centrifugal pump. After resuming flow to the patient, the flow was 3.9 L/min at 3500 RPMs. The second step in the troubleshooting process was checking to make sure the flow probe was accurately reading. The flow was measured via the LivaNova Sorin S5 built-in flow probe that was placed pre-oxygenator for the most accurate total flow measurements. To quickly verify accurate flow measurement, a Centrimag (Abbott, Abbott Park, IL) was brought into the operating room. The team disengaged the flow probe from the cardiopulmonary bypass machine and placed the flow probe from the Centrimag onto the CPB circuit and flows were correlating at 3.9 L/min at 3500 RPMs. The next step was to check the placement of the aortic cannula. Due to a discrepancy of about 20 mmHg in the aortic line pressure and the patient’s right radial artery pressure before bypass, it was believed it was in our best interest to check cannula placement. At this time, the line pressures were arterial line resistance 153 mmHg, radial arterial line 70 mmHg, and femoral arterial line 71 mmHg. At this time, the heart was arrested, and the line pressure was laminar flow. The transducers on the CPB circuit were zeroed prior to the start of the case during morning pump checks. We started with readjusting the arterial cannula and checking for an aortic dissection under a transesophageal echocardiogram. After it was determined that the cannula was still in the true lumen of the aorta and there was no new dissection, flows dropped gradually over about 10–15 min to 2 L/min. As a final troubleshooting step, the oxygenator purge line was used to check the pre-membrane pressure of the oxygenator. On the Medtronic Affinity Fusion, the purge line is a pre-membrane purge line and therefore could be used to read pre-oxygenator pressure. The purge line was connected directly to the arterial transducer on the CPB pump. With the transducer turned off to the circuit and onto the oxygenator purge line the pressure was reading >550 mmHg. At this point, the perfusion team decided to change the oxygenator on the circuit to a new Affinity Fusion oxygenator. The oxygenator was primed and changed in the usual fashion without incident with the changeout process was completed in less than 1 min. With the new oxygenator connected, the flows were increased to 5.91 L/min at 3300 RPM with 229 mmHg resistance, and the radial artery blood pressure was 69 mmHg.

Five minutes following the replacement of the oxygenator, the flows decreased to 5.29 L/min with maximum RPMs of 3500, and over the next 15 min, the flows continued to steadily decrease to 4.50 L/min although the RPMs remained high at 3497. At this point, the patient was fully cooled to 22.1 °C and DHCA with RCP was initiated and cardiopulmonary bypass was turned off. The perfusion team decided to exchange the oxygenator again during the DHCA period. For the second oxygenator exchange, it was decided to use a Terumo FX25 (Terumo Cardiovascular, Ann Arbor, MI) oxygenator instead of a Medtronic Affinity Fusion.

After completion of the hemiarch replacement, CPB was resumed, and the patient was rewarmed. With the Terumo FX25 oxygenator, the flows increased to 6.15 L/min at 2801 RPMs with 182 mmHg resistance. The blood pressure was 73 mmHg. At 1016 another TEG was completed. At this time the platelet count was 17,000/mL. During the case, the TEG drifted more towards normal throughout the case and ended at 39.5 mm post bypass Blood gases were monitored through the rest of the case with no significant changes. Likewise, for the remainder of the case, the flows did not drop below 6.0 L/min. Following CPB, 200 mg protamine was given at 1105, and ACT at 1110 was 126.

## Comment

Oxygenator HPE is a rare event that is associated with an incidence of approximately 0.4–2.3% of cases [3]. In this case, the patient had a positive surgical outcome as is evident by a definitive aortic repair with next-day extubation and no documented neurological deficits. This successful result can be attributed to the quick intervention of the perfusion and surgical team. In retrospect and after the literature review, more troubleshooting should have been attempted before the oxygenator changeout. Epoprostenol administration and hemodilution could have been attempted to prevent the necessity for oxygenator changeout. In this case, however, rewarming the patient would not have been possible due to the necessity for DHCA. These troubleshooting steps were not part of our policy and procedure manual and were therefore not utilized. As a direct result of this case, epoprostenol administration, hemodilution, and patient rewarming have been added to our policy and procedures for HPE.

The calculated lean body mass for the patient was calculated to be 105 kg. This was not part of our standard of care to be calculated before the case. However, following the case, the proper flows according to the ELBM were calculated. To acquire a minimum of 2.0 CI, flows should be 4.6 L/min. With this now in mind, the patient only had flows below 4.6 L/min directly before the troubleshooting began. This supported our decision to only include one oxygenator. During the case, the perfusion team was unaware of the platelet decrease. The TEG showed at 0755 the platelet count was 214,000. This was a 13.0% decrease in platelet count from the preoperative labs to the first blood draw in the operating room. After another 261 min, the platelet count decreased by another 92.1%, specifically to 17,000. The observed decrease in platelet count may be explained by platelet aggregation at the oxygenator. This phenomenon is not a new discovery; platelets are known to attach to the surface area of the oxygenator membranes [4]. The observed decrease in platelet count and plausible platelet aggregation is a potential leading cause of the HPE and the decreased flow of blood through the oxygenator.

The major difference between the Medtronic and the Terumo oxygenators was the blood flow path (Table 1). The Medtronic oxygenator which has a radial flow design was first used in the circuit for this case. The inlet of the Medtronic is above the outlet; therefore, blood must flow down through the oxygenator, parallel to the fiber bundle. Because of this longer flow path through the fiber bundle, the Affinity pump must pump against increased resistance to begin with.

**Table 1:** Distinct differences and similarities of the Medtronic Affinity NT Fusion and Terumo FX25 Oxygenator.

	Medtronic fusion oxygenator	Terumo FX25 oxygenator
Blood flow	Radially	Horizontally
Arterial filter size	25 μm	32 μm
Prime volume	260 mL	260 mL
Surface area of fibers	2.5 m^2^	2.5 m^2^
Material	Microporous Polypropylene	Polyester Screen type
Flow range	1–7 L/min	0.5–7 L/min
Maximum blood pressure	750 mmHg	1,000 mmHg
Surface coating	Balance^TM^ BioSurface	X-Coating
Heat exchanger	Plastic	Stainless Steel

This blood flow path in combination with the patient’s large BSA may be a plausible reason for the observance of HPE in this case. In circuits with oxygenators that feature lower resistance, HPE may not have been noticed as the RPMs could be turned up to a flow that would support the patient. Following the incidence of HPE in this case, the Medtronic oxygenator was changed to a Terumo transverse flow oxygenator. The inlet of this model is on the side and the outlet is at 90° to the inlet on the front of the device. Additionally, blood flows perpendicular to the oxygenator and therefore has a shorter flow path across the fiber bundle. This leads to less resistance from this oxygenator as evidenced by the ability to generate a higher flow at lower RPMs once the oxygenator was changed to the Terumo FX25. Since studies in HPE have been completed on roller pumps, it is unclear whether these steps will resolve the issue completely in cases with centrifugal pumps, but they are nonetheless worth attempting if oxygenator changeout can be avoided [2]. While oxygenator changeouts due to HPE are not unique, it was notable that two Medtronic Affinity NT Fusion oxygenators had to be changed out in this case.

## Data Availability

No new data was created or analyzed for this study.
