# Corrigendum to: High pressure excursion in a radial design oxygenator

**DOI:** 10.1051/ject/2025054

**Published:** 2026-06-19

**Authors:** Ashley Svec, Tyler Eadie, Brandon D’Aloiso, Peter Arlia

**Affiliations:** 1 UPMC Presbyterian Perfusion 200 Lothrop Street Pittsburgh PA 15213 USA

Paragraph 3 of the “Description” section should have read: “The Anesthesia team gave 40,000 units heparin”. It was previously incorrectly stated that only 4,000 units of heparin were given.

